# Hospital Utilization, Costs, and Mortality for Adults With Multiple Chronic Conditions, Nationwide Inpatient Sample, 2009

**DOI:** 10.5888/pcd10.120292

**Published:** 2013-04-25

**Authors:** Claudia A. Steiner, Bernard Friedman

**Affiliations:** Author Affiliations: Bernard Friedman, Agency for Healthcare Research and Quality, Rockville, Maryland.

## Abstract

**Objective:**

Our objective was to provide a national estimate across all payers of the distribution and cost of selected chronic conditions for hospitalized adults in 2009, stratified by demographic characteristics.

**Analysis:**

We analyzed the Nationwide Inpatient Sample (NIS), the largest all-payer inpatient database in the United States. Use, cost, and mortality estimates across payer, age, sex, and race/ethnicity are produced for grouped or multiple chronic conditions (MCC). The 5 most common dyads and triads were determined.

**Results:**

In 2009, there were approximately 28 million adult discharges from US hospitals other than those related to pregnancy and maternity; 39% had 2 to 3 MCC, and 33% had 4 or more. A higher number of MCC was associated with higher mortality, use of services, and average cost. The percentages of Medicaid, privately insured patients, and ethnic/racial groups with 4 or more MCC were highly sensitive to age.

**Summary:**

This descriptive analysis of multipayer inpatient data provides a robust national view of the substantial use and costs among adults hospitalized with MCC.

## Introduction

The prevalence of adults with more than 1 coexisting chronic condition, often referred to as multiple chronic conditions (MCC), is large and growing in the United States. As the US population ages, the Partnership for Solutions projects that by 2020, one-quarter of Americans will live with MCC (1). Consequences of multiple chronic conditions include impacts on health, quality, delivery of care, and cost. Nationwide, expenses for hospital inpatient care remain the largest component of total health care expenditures. A previous study of hospitalizations using a national all-payer database demonstrated that the number of chronic conditions independently influences hospital costs. Patients with complex illness, defined as 3 or more chronic conditions, were found to have a disproportionately large association with hospital cost per year (2) Another study using hospitalizations from statewide databases that support readmission analyses demonstrated that the likelihood of a readmission was related to the complexity of chronic illness as measured by the number of different chronic conditions (3).

The primary objective of this study is to describe the distribution of multiple chronic conditions among patients hospitalized in US community hospitals in 2009. The study includes all payers, including private, public, and uninsured. We also describe hospitalizations subclassified by patient’s payer or by race/ethnicity, within age groups and by sex. In addition, we describe the most common dyads and triads of chronic conditions by demographic characteristics.

## Analysis

The data source for this study is the Nationwide Inpatient Sample (NIS) of the Healthcare Cost and Utilization Project (HCUP), the largest all-payer inpatient database in the United States (4). The NIS is designed to approximate a 20% sample of US community hospitals, defined by the American Hospital Association as “all nonfederal, short-term, general, and other specialty hospitals, excluding hospital units of institutions.” The NIS hospital sample is drawn from states participating in HCUP. For 2009, these 44 states comprise more than 95% of the US population. This universe of US community hospitals is divided into strata by 5 hospital characteristics: ownership/control, number of beds, teaching status, urban/rural location, and US region. The NIS is a stratified probability sample of hospitals; sampling probabilities are proportional to the number of US community hospitals in each stratum. The 2009 NIS includes all discharge data from 1,050 hospitals that were selected for the sample, a total of 7,810,762 unweighted discharges. Sample weights are provided to produce national estimates.

The study population is restricted to adult patients aged 18 or older admitted for diagnoses other than pregnancy and maternity. The expected payers are defined hierarchically by using primary and secondary expected payer as Medicare, then Medicaid, privately insured, and uninsured. Some “other” categories that include private insurers and public funding are not uniformly reported, so they are included in the privately insured group. The age groups are 18 to 44 years, 45 to 64 years, and 65 or older. Race and ethnicity are defined as white, black, Hispanic, Asian/Pacific Islander, and Native American.

### Enhancement of hospital sample for race/ethnicity coding

 Some states and hospitals do not provide patient race or ethnicity on each discharge record. Therefore, an internal Agency for Healthcare Research and Quality (AHRQ)-enhanced version of the NIS was created to produce robust national estimates stratified by race and ethnicity. For hospitals that do not provide race/ethnicity, the enhanced database randomly selected additional hospitals in the same stratum without replacement or duplication, attempting to reach the 20% target of hospitals in the stratum. This method of preparing data are used in the National Hospital Disparities Report (5,6).

### Calculation of the number of chronic conditions

We used the set of chronic conditions developed by the Department of Health and Human Services (HHS) Interagency Workgroup on MCC and the Office of the Assistant Secretary of Health (7). Highly related diagnoses of the same condition were grouped together using an established Clinical Classification System (CCS) (8). The CCS categories were matched to the established set of chronic conditions; an exception was made for Autism Spectrum Disorder, for which individual ICD-9-CM codes were used. Steps were taken to avoid overcounting the number of 15 conditions reported on a single discharge summary. No one CCS category was counted more than once. In addition, we grouped clusters of CCS categories for highly related conditions (eg, 2 CCS categories for diabetes are clustered together). No cluster is counted more than once. The criteria used to identify the 15 chronic conditions are provided in an Appendix. Chronic conditions on each discharge record were counted and grouped into 3 categories (0-1, 2–3, and 4 or more). Multiple chronic conditions (MCC) are defined as 2 or more chronic conditions.

### Cost and mortality

Cost is an estimate of resources used in production of service and includes direct hospital costs without physician fees. All-payer, inpatient cost-to-charge ratios are constructed from the hospital’s accounting data for 2009 as reported to the Centers for Medicare and Medicaid Services (CMS). For 10% to 15% of hospitals, cost-to-charge ratios are estimated by imputation within state by hospital characteristics (9). Mortality rates represent only deaths in the hospital.

## Results

We calculated a national estimate of the overall distribution of multiple chronic conditions for adult discharges by age group, expected primary payer, and sex (Table 1). Each column shows several use and cost items for the number of chronic conditions (0-1, 2–3, 4 or more), including the mortality rate and mean length of stay, and cost. Roughly one-third of discharges were in the highest MCC grouping (4 or more). The mortality rate was higher for adults discharged with 4 or more MCC compared with that of adults with 0–1 chronic conditions (3.1% vs 1.9%); there was a longer length of stay and a 9% higher cost per discharge.

**Table 1:** Adult Hospital Discharges, by Number of Chronic Conditions Across Payer, Sex, Age, Race/Ethnicity, Nationwide Inpatient Sample, 2009

Discharge Characteristic	No. of Chronic Conditions			
	0 or 1	2 or 3	≥4	All
**All Adult Discharges**				
Discharges, n (%)^a^	8,167,314 (28.81)	10,929,300 (38.56)	9,252,415 (32.64)	28,349,029 (100.00)
Mortality rate	0.02	0.03	0.03	0.03
Mean length of stay, d	4.46	5.21	5.42	5.06
Mean charge, $	35,385.98	37,602.67	38,672.55	37,311.28
Mean cost, $	10,544.91	11,180.93	11,480.79	11,095.01
**Payer**				
**Medicare**				
n (%)	2,204,737 (14.54)	6,033,738 (39.80)	6,922,039 (45.66)	15,160,515 (100.00)
Mortality rate	0.04	0.03	0.03	0.03
Mean length of stay, d	5.62	5.50	5.51	5.52
Mean charge, $	39653.15	38123.91	37786.48	38193.47
Mean cost, $	11,922.59	11,394.49	11,284.88	11,421.66
**Medicaid**				
n (%)	1,181,760 (37.01)	1,280,715 (40.11)	730,609 (22.88)	3,193,083 (100.00)
Mortality rate	0.02	0.02	0.02	0.02
Mean length of stay, d	5.26	5.95	5.89	5.68
Mean charge, $	37,242.88	36,467.74	39,491.62	37,445.82
Mean cost, $	10,634.06	10,398.02	11,244.41	10,678.85
**Private**				
n (%)	3,875,344 (47.10)	2,977,623 (36.19)	1,374,750 (16.71)	8,227,717 (100.00)
Mortality rate	0.01	0.02	0.02	0.02
Mean length of stay, d	3.72	4.45	4.82	4.17
Mean charge, $	34,257.60	38,879.88	43,095.20	37,397.61
Mean cost, $	10,277.17	11,626.10	12,693.26	11,166.47
**Self-pay**				
n (%)	905,473 (51.22)	637,224 (36.05)	225,017 (12.73)	1,767,713 (100.00)
Mortality rate	0.01	0.02	0.02	0.01
Mean length of stay, d	3.75	4.49	4.97	4.17
Mean charge, $	27,386	29,088.97	36,492.51	29,160.09
Mean cost, $	8,218.75	8,691.24	10,945.66	8,736.48
**Sex**				
**Men**				
n (%)	3,582,447 (27.41)	5,072,042 (38.80)	4,417,230 (33.79)	13,071,719 (100.00)
Mortality rate	0.02	0.02	0.03	0.02
Mean length of stay, d	4.19	5.13	5.45	4.95
Mean charge, $	38,860.38	40,070.49	41,303.33	40,153.68
Mean cost, $	11,524.23	11,873.24	12,207.98	11,890.22
**Women**				
n (%)	4,569,669 (29.89)	5,871,812 (38.41)	4,844,323 (31.69)	15,285,805 (100.00)
Mortality rate	0.02	0.02	0.03	0.02
Mean length of stay, d	4.19	5.13	5.45	4.95
Mean charge, $	32,646.41	35,449.35	36,266.74	34,868.34
Mean cost, $	9,790.54	10,583.73	10,820.74	10,421.11
**Age**				
**18–44 y**				
n (%)	3,419,009 (63.36)	1,624,350 (30.10)	352,970.50 (6.54)	5,396,330 (100.00)
Mortality rate	0.01	0.01	0.01	0.01
Mean length of stay, d	3.91	5.03	5.20	4.33
Mean charge, $	30,804.92	30,364.65	34,111.91	30,910.92
Mean cost, $	9,142.41	9,025.33	10,080.57	9,168.51
**45–64 y**				
n (%)	2,992,037 (31.60)	3,987,333 (42.11)	2,488,444 (26.28)	9,467,814 (100.00)
Mortality rate	0.02	0.02	0.02	0.02
Mean length of stay, d	4.50	5.05	5.26	4.93
Mean charge, $	37,773.66	39,377.85	40,983.65	39,292.56
Mean cost, $	11,299.41	11,710.14	12,107.17	11,684.60
**≥65 y**				
Discharges, n (%)	1,774,032 (13.11)	5,334,250 (39.43)	6,420,323 (47.46)	13,528,605 (100.00)
Mortality rate	0.05	0.04	0.04	0.04
Mean length of stay, d	5.44	5.38	5.50	5.44
Mean charge, $	40,068.53	38,467.4	38,022.14	38,467.45
Mean cost, $	11,974.46	11,444.67	11,317.35	11,454.16
**Race/Ethnicity**				
**White**				
n (%)	5,666,925 (27.62)	7,942,189 (38.70)	6,911,652 (33.68)	20,520,766 (100.00)
Mortality rate	0.02	0.03	0.03	0.03
Mean length of stay, d	4.37	5.06	5.30	4.95
Mean charge, $	34,050.26	35,737.8	36,542.4	35,541.02
Mean cost, $	10,576.29	11,075.09	11,266.23	11,001.25
**Black**				
n (%)	1,045,058 (27.01)	1,546,737 (39.98)	1,277,201 (33.01)	3,868,996 (100.00)
Mortality rate	0.02	0.02	0.02	0.02
Mean length of stay, d	4.99	5.74	5.81	5.56
Mean charge, $	35,929.6	38,679.35	39,139.03	38,087.77
Mean cost, $	10,114.23	10,877.67	11,276.55	10,802.81
**Hispanic**				
n (%)	890,011 (37.49)	859,422 (36.20)	624,850 (26.32)	2,374,283 (100.00)
Mortality rate	0.02	0.02	0.03	0.02
Mean length of stay, d	4.37	5.37	5.74	5.10
Mean charge, $	39,520.76	45,699.29	51,147.17	44,807.28
Mean cost, $	10,241.91	11,319.79	12,467.65	11,215.95
**Asian/Pacific Islander**				
n (%)	162,048 (31.36)	196,067 (37.94)	158,688 (30.71)	516,804 (100.00)
Mortality rate	0.03	0.04	0.04	0.03
Mean length of stay, d	4.80	5.68	5.92	5.48
Mean charge, $	47,747.97	57,105.99	64,097.02	56,257.44
Mean cost, $	12,455.85	14,280.97	15,380.64	14,035.66
**Native American**				
n (%)	61,241.47 (31.16)	76,597.34 (38.97)	58,721.93 (29.87)	196,560.7 (100.00)
Mortality rate	0.02	0.02	0.03	0.02
Mean length of stay, d	4.35	5.05	5.03	4.83
Mean charge, $	30,114.33	32,991.89	37,077.33	33,314.41
Mean cost, $	10,368.74	11,160.54	11,499.38	11,014.92

a The number of all discharges within the columns of each detailed breakdown section of the table may not add precisely to all discharges in the first line of the table because of missing data on national estimates within each section. For more information on sampling variation and missing data for particular variables, consult www.hcupnet.ahrq.gov.

Medicare covers 53.7% of all the discharges and has a higher share of the discharges with 4 or more MCC (74.8%). Fewer than half (46%) of Medicare discharges had 4 or more chronic conditions. For privately insured patients, only 16.7% had 4 or more chronic conditions. Men and women both had about one-third of discharges with 4 or more chronic conditions. The differences by age are striking. Only 6.5% of discharges aged 18 to 44 years had 4 or more chronic conditions, whereas adults aged 65 or older had rates similar to those of the entire Medicare population (47.5% with 4 or more chronic conditions). We found small differences in the distribution of chronic conditions by racial/ethnic groups. The proportion of adults discharged with 4 or more chronic conditions was lowest for Hispanics (26.3%) and highest for whites (33.7%). Asian/Pacific Islanders had the highest mortality regardless of number of chronic conditions, and the highest costs per case ($14,000) compared with $11,000 for all groups combined.

The distribution of discharges for different payer categories were nested within age and sex (Table 2). In this context, substantial differences can be seen in the Medicaid-covered population by age group. Younger adults covered by Medicaid have a relatively low percentage of adults with 4 or more MCC (9% for men, 7.7% for women). The percentage for each sex rises to about 32% for adults aged 45 through 64 and then to 42% for adults aged 65 or older. Uninsured adults and adults with private payers had a lower percentage of discharges with 4 or more MCC across all age groups and each sex.

**Table 2:** Adults Discharged From US Hospitals by Payer Within Age and Sex, Nationwide Inpatient Sample, 2009

Discharge Characteristic	No. of Chronic Conditions			
	0 to 1	2 to 3	≥4	All
**Men aged 18–44 y**				
**Medicare**				
n (%)	108,888 (34.78)	145,761 (46.56)	58,433 (18.66)	313,082 (100.00)
Mortality rate	0.01	0.01	0.01	0.01
Mean length of stay, d	6.31	6.30	5.79	6.21
Mean charge, $	36,282.56	33,043.79	34,346.26	34,413.36
Mean cost, $	10,765.5	9,784.298	10,156.62	10,195.06
**Medicaid**				
n (%)	312,401 (51.81)	236,287 (39.18)	54,321 (9.01)	603,009 (100.00)
Mortality rate	0.01	0.01	0.01	0.01
Mean length of stay, d	3.78	4.49	4.52	4.01
Mean charge, $	34,158.33	32,689.01	38,973.72	33,976.47
Mean cost, $	10,066.80	9,690.28	11,398.59	10,024.63
**Private**				
n (%)	758,767 (68.21)	303,074 (27.25)	50,517 (4.54)	1,112,358 (100.00)
Mortality rate	0.01	0.01	0.01	0.01
Mean length of stay, d	3.78	4.49	4.52	4.01
Mean charge, $	34,158.33	32,689.01	38,973.72	33,976.47
Mean cost, $	10066.80	9690.28	11398.59	10024.63
**Self-pay**				
n (%)	351,196 (64.58)	166,152 (30.55)	26,455 (4.86)	543,804 (100.00)
Mortality rate	0.01	0.01	0.01	0.01
Mean length of stay, d	3.65	4.37	4.39	3.91
Mean charge, $	25,756.81	23,822.86	30,858.46	25,414.02
Mean cost, $	7,793.37	7,160.63	9,027.34	7,660.02
**Men aged 45–64 y**				
**Medicare**				
n (%)	203,301 (17.84)	467,741 (41.04)	468,674 (41.12)	1,139,716 (100.00)
Mortality rate	0.03	0.02	0.02	0.02
Mean length of stay, d	6.55	6.09	5.56	5.95
Mean charge, $	44,284.56	40,537.61	39,541.04	40,796.60
Mean cost, $	13,245.30	11,960.11	11,666.46	12,068.74
**Medicaid**				
n (%)	182,430 (23.77)	343,745 (44.80)	241,169 (31.43)	767,344 (100.00)
Mortality rate	0.03	0.02	0.02	0.02
Mean length of stay, d	6.75	6.41	5.95	6.35
Mean charge, $	45,922.70	40,771.91	40,881.27	42,030.43
Mean cost, $	13,085.48	11,751.80	11,711.18	12,056.01
**Private**				
n (%)	813767 (33.87)	1051276 (43.76)	537256 (22.36)	2402299 (100.00)
Mortality rate	0.02	0.02	0.01	0.02
Mean length of stay, d	4.24	4.35	4.64	4.38
Mean charge, $	40,495.69	42,715.50	46,321.88	42,767.70
Mean cost, $	12,150.26	12,752.72	13,698.33	12,759.49
**Self-pay**				
n (%)	166,307 (35.49)	205,565 (43.87)	96,733 (20.64)	468,605 (100.00)
Mortality rate	0.02	0.02	0.02	0.02
Mean length of stay, d	4.56	4.78	5.01	4.75
Mean charge, $	31,775.72	33,344.77	39,030.38	33,961.61
Mean cost, $	9,579.73	9,992.05	11,507.92	10,158.64
**Men aged ≥65 y**				
**Medicare**				
n (%)	665,135 (12.62)	2,014,678 (38.24)	2,588,693 (49.14)	5,268,507 (100.00)
Mortality rate	0.05	0.04	0.04	0.04
Mean length of stay, d	5.81	5.48	5.43	5.50
Mean charge, $	43,426.57	41,082.58	39,831.74	40,765.79
Mean cost, $	13,121.63	12,303.19	11,882.03	12,200.22
**Medicaid**				
n (%)	14,603 (16.51)	37,212 (42.07)	36,630 (41.42)	88,445 (100.00)
Mortality rate	0.06	0.05	0.04	0.05
Mean length of stay, d	7.20	7.07	7.21	7.15
Mean charge, $	52,644.60	48,780.02	48,914.96	49,474.11
Mean cost, $	14,215.67	13,230.12	13,480.00	13,496.22
**Private**				
Discharges, n (%)	76,922 (16.40)	189,141 (40.31)	203,115 (43.29)	469,177 (100.00)
Mortality rate	0.08	0.06	0.05	0.06
Mean length of stay, d	5.30	5.13	5.21	5.19
Mean charge, $	45,328.43	44,083.07	43,782.92	44,158.40
Mean cost, $	13,026.96	12,629.63	12,440.45	12,613.35
**Self-pay**				
n (%)	7,836 (21.69)	14,798 (40.95)	13,501 (37.36)	36,136 (100.00)
Mortality rate	0.108	0.06	0.06	0.07
Mean length of stay, d	5.58	5.54	6.17	5.79
Mean charge, $	36,910.76	39,488.91	39,595.57	38,970.75
Mean cost, $	11,220.25	12,522.91	12,651.25	12,288.72
**Women aged 18–44 y**				
**Medicare**				
n (%)	119,666 (38.58)	140,524 (45.31)	49,967 (16.11)	310,157 (100.00)
Mortality rate	0.01	0.01	0.01	0.01
Mean length of stay, d	5.49	5.90	5.54	5.69
Mean charge, $	32,332.45	33,295.78	33,724.04	32,992.90
Mean cost, $	9,748.48	9,783.22	9,955.79	9,797.59
**Medicaid**				
n (%)	4,492,580 (58.93)	278,647 (33.34)	64,634 (7.73)	835,860 (100.00)
Mortality rate	0.01	0.01	0.01	0.01
Mean length of stay, d	4.24	5.31	5.32	4.68
Mean charge, $	28,398.89	27,609.89	30,565.52	28,303.17
Mean cost, $	8,376.36	8,193.98	9,117.12	8,372.78
**Private**				
n (%)	1,048,248 (75.45)	303,657 (21.86)	37,472 (2.70)	1,389,376 (100.00)
Mortality rate	0.00	0.01	0.01	0.00
Mean length of stay, d	3.21	4.24	4.47	3.47
Mean charge, $	27,901.65	29,707.63	34,695.92	28,478.33
Mean cost, $	8,312.62	8,834.00	10,243.32	8,478.28
**Self-pay**				
n (%)	248,365 (69.35)	96,378 (26.91)	13,406 (2.70)	358,149 (100.00)
Mortality rate	0.00	0.01	0.01	0.01
Mean length of stay, d	3.27	4.10	4.40	3.54
Mean charge, $	22,693.59	22,365.63	29,013.77	22,842.02
Mean cost, $	6,926.04	6,738.00	8,623.15	6,938.96
**Women aged 45–64 y**				
**Medicare**				
n (%)	224,202 (20.00)	474,563 (42.34)	422,019 (37.65)	1,120,783 (100.00)
Mortality rate	0.02	0.02	0.02	0.02
Mean length of stay, d	6.00	5.84	5.54	5.76
Mean charge, $	39,567.05	37,455.52	37,136.58	37,758.12
Mean cost, $	11,987.25	11,229.84	11,025.40	11,304.51
**Medicaid**				
n (%)	210,734 (24.40)	373,959 (43.30)	278,978 (32.30)	863,671 (100.00)
Mortality rate	0.02	0.02	0.01	0.02
Mean length of stay, d	5.75	5.85	5.70	5.78
Mean charge, $	39,336.88	37,011.21	36,886.57	37,538.15
Mean cost, $	11,395.97	10,680.02	10,791.38	10,890.61
**Private**				
n (%)	1,080,447 (44.02)	988,812 (40.29)	385,165 (15.69)	2,454,423 (100.00)
Mortality rate	0.01	0.01	0.01	0.01
Mean length of stay, d	3.73	4.31	4.63	4.11
Mean charge, $	34,117.03	37,245.12	39,641.79	36,240.15
Mean cost, $	10,300.35	11,264.37	11,941.66	10,945.07
**Self-pay**				
n (%)	143,013 (39.43)	154,460 (42.59)	65,219 (17.98)	362,692 (100.00)
Mortality rate	0.02	0.01	0.02	0.02
Mean length of stay, d	3.97	4.49	4.73	4.33
Mean charge, $	27,568.26	29,511.21	33,909.31	29,536.17
Mean cost, $	8,578.13	9,032.98	10,415.54	9,102.30
**Women aged ≥65**				
**Medicare**				
n (%)	936,232 (13.35)	2,873,933 (40.97)	3,205,064 (45.69)	7,015,230 (100.00)
Mortality rate	0.04	0.03	0.03	0.03
Mean length of stay, d	5.45	5.30	5.46	5.39
Mean charge, $	37,312.62	34,902.96	34,767.87	35,163.93
Mean cost, $	11,430.65	10,588.52	10,490.42	10,656.53
**Medicaid**				
n (%)	21,863 (14.60)	62,869 (41.97)	65,057 (43.43)	149,789 (100.00)
Mortality rate	0.05	0.04	0.03	0.04
Mean length of stay, d	6.36	6.38	6.99	6.65
Mean charge, $	42,907.69	40,707.18	42,918.38	41,988.29
Mean cost, $	11,977.38	11,338.54	11,972.17	11,706.85
**Private**				
n (%)	83,905 (18.33)	197,581 (43.17)	176,220 (38.50)	457,706 (100.00)
Mortality rate	0.07	0.05	0.05	0.06
Mean length of stay, d	4.94	5.01	5.19	5.06
Mean charge, $	36,988.99	37,399.27	37,110.41	37,212.95
Mean cost, $	10,922.90	10,808.53	10,692.03	10,784.93
**Self-pay**				
n (%)	9,699 (23.06)	18,097 (43.03)	14,260 (33.91)	42,056 (100.00)
Mortality rate	0.07	0.06	0.05	0.06
Mean length of stay, d	6.40	5.48	6.20	5.94
Mean charge, $	31,791.20	32,658.91	34,218.72	32,988.01
Mean cost, $	10,023.66	10,349.79	11,124.45	10,537.39

We analyzed differences in distribution and outcome by racial/ethnic groups nested within age groups and sex (Table 3). In younger age groups, a higher proportion of black men discharged have 4 or more MCC than do any other racial/ethnic group. This same was true of black women aged 18 through 44. Differences among adults by race and ethnicity are hidden when ages are combined.

**Table 3:** Adults Discharged From US Hospitals, by Race/Ethnicity Within Age Groups and Sex, Nationwide Inpatient Sample, 2009

Discharge Characteristic	No. of Chronic Conditions				
	0 to 1	2 to 3	≥4		All
**Men aged 18–44 y**					
**White**					
n (%)	917,075 (60.83)	492,730 (32.68)	97,880 (6.49)	1,507,685 (100.00)	
Mortality rate	0.01	0.01	0.01	0.01	
Mean length of stay, d	4.10	4.94	5.06	4.44	
Mean charge, $	32,308.69	28,636.78	33,143.72	31,161.70	
Mean cost, $	9,937.46	89,10.26	10,198.32	9,618.37	
**Black**					
n (%)	238,958 (49.93)	181,964 (38.02)	57,708 (12.06)	478,630 (100.00)	
Mortality rate	0.01	0.01	0.01	0.01	
Mean length of stay, d	5.03	5.65	5.51	5.33	
Mean charge, $	34,340.21	31,673.97	34,212.79	33,310.18	
Mean cost, $	9,709.54	9,155.93	10,078.22	9,543.23	
**Hispanic**					
n (%)	22,8901 (65.46)	99,352 (28.41)	21,411 (6.12)	349,664 (100.00)	
Mortality rate	0.01	0.01	0.01	0.01	
Mean length of stay, d	4.56	5.59	5.33	4.90	
Mean charge, $	40,675.98	40,163.08	43,418.28	40,697.90	
Mean cost, $	10,928.28	10,542.99	11,013.78	10,824.10	
**Asian/Pacific Islander**					
n (%)	26,604 (65.46)	11,263 (27.71)	2,777 (6.83)	40,644 (100.00)	
Mortality rate	0.01	0.02	0.01	0.01	
Mean length of stay, d	4.94	5.96	5.49	5.26	
Mean charge, $	46,928.06	51,705.31	55,389.21	48,806.68	
Mean cost, $	12,290.35	13,744.53	13,736.64	12,786.57	
**Native American**					
n (%)	12,665 (56.31)	8,040 (35.75)	1,785 (7.94)	22,490 (100.00)	
Mortality rate	0.01	0.01	0.02	0.01	
Mean length of stay, d	4.24	4.99	4.72	4.55	
Mean charge, $	29,490.33	26,213.56	33,706.71	28,648.17	
Mean cost, $	9,733.02	8,618.44	10,976.53	9,431.61	
**Men aged 45–64 y**					
**White**					
n (%)	964,565 (29.61)	1,398,301 (42.92)	895,219 (27.48)	3,258,085 (100.00)	
Mortality rate	0.02	0.02	0.02	0.02	
Mean length of stay, d	4.70	4.90	5.07	4.89	
Mean charge, $	39,740.38	40,268.24	41,721.88	40,511.04	
Mean cost, $	12,290.35	12,403.48	12,768.23	12,470.13	
**Black**					
n (%)	156,756 (20.67)	339,238 (44.73)	262,498 (34.61)	758,492 (100.00)	
Mortality rate	0.02	0.02	0.02	0.02	
Mean length of stay, d	5.74	5.74	5.62	5.70	
Mean charge, $	42,500.38	39,497.04	40,095.68	40,324.30	
Mean cost, $	11,874.92	11,212.07	11,508.44	11,451.44	
**Hispanic**					
n (%)	124,063 (30.99)	168,557 (42.10)	107,739 (26.91)	400,359 (100.00)	
Mortality rate	0.02	0.02	0.02	0.02	
Mean length of stay, d	5.10	5.48	5.58	5.39	
Mean charge, $	46,103.51	48,804.00	52,895.88	49,065.06	
Mean cost, $	11,925.25	12,216.00	13,132.87	12,372.10	
**Asian/Pacific Islander**					
n (%)	23,944 (30.84)	32,410 (41.75)	21,279 (27.41)	77,633 (100.00)	
Mortality rate	0.03	0.03	0.02	0.03	
Mean length of stay, d	5.32	5.58	5.84	5.57	
Mean charge, $	56,263.83	62,086.23	71,098.07	62,681.37	
Mean cost, $	14,496.26	15,730.86	17,496.28	15,817.94	
**Native American**					
n (%)	9,690 (26.63)	15,708 (43.18)	10,983 (30.19)	36,381 (100.00)	
Mortality rate	0.03	0.02	0.01	0.02	
Mean length of stay, d	5.09	4.92	4.79	4.93	
Mean charge, $	36,101.52	35,412.00	39,829.43	36,931.24	
Mean cost, $	12,672.81	11,841.69	12,230.61	12,179.48	
**Men aged ≥65 y**					
**White**					
n (%)	594,416 (12.67)	1,767,324 (37.66)	2,331,641 (49.68)	4,693,381 (100.00)	
Mortality rate	0.05	0.04	0.04	0.04	
Mean length of stay, d	5.45	5.32	5.35	5.35	
Mean charge, $	41,150.03	39,783.78	38,603.28	39,372.19	
Mean cost, $	12,690.98	12,229.29	11,821.99	12,086.05	
**Black**					
n (%)	52,675 (10.77)	187,401 (38.31)	249,085 (50.92)	489,161 (100.00)	
Mortality rate	0.07	0.04	0.04	0.04	
Mean length of stay, d	7.27	6.45	6.14	6.38	
Mean charge, $	52,723.93	46,092.80	41,837.77	44,645.21	
Mean cost, $	14,513.92	12,679.25	12,011.12	12,537.51	
**Hispanic**					
n (%)	54,561 (14.78)	144,823 (39.23)	169,737 (45.98)	369,121 (100.00)	
Mortality rate	0.05	0.04	0.04	0.04	
Mean length of stay, d	5.76	5.82	6.03	5.91	
Mean charge, $	52,214.78	53,118.44	56,508.38	54,535.47	
Mean cost, $	12,531.53	12,715.68	13,583.41	13,085.42	
**Asian/Pacific Islander**					
n (%)	16,563 (14.09)	47,631 (40.53)	53,322 (45.37)	11,7517 (100.00)	
Mortality rate	0.06	0.05	0.05	0.05	
Mean length of stay, d	6.14	6.13	6.08	6.11	
Mean charge, $	62,512.13	64,955.69	68,702.57	66,278.41	
Mean cost, $	15,711.12	15,857.96	16,261.18	16,016.96	
**Native American**					
n (%)	4,597 (14.04)	12,286 (37.53)	15,855 (48.43)	32,738 (100.00)	
Mortality rate	0.05	0.04	0.03	0.04	
Mean length of stay, d	5.22	5.41	5.00	5.18	
Mean charge, $	38,101.53	39,401.45	41,222.76	40,099.73	
Mean cost, $	12,964.16	13,579.89	12,647.02	13,041.83	
**Women aged 18–44 y**					
**White**					
n (%)	1,173,549 (66.97)	491,581 (28.05)	87,176 (4.97)	1,752,306 (100.00)	
Mortality rate	0.00	0.01	0.01	0.00	
Mean length of stay, d	3.45	4.63	4.88	3.85	
Mean charge, $	26,383.05	26,096.09	30,033.49	26,483.96	
Mean cost, $	8,282.15	8,209.02	9,332.88	8,313.86	
**Black**					
n (%)	329,648 (57.58)	187,273 (32.71)	55,613 (9.71)	572,533 (100.00)	
Mortality rate	0.00	0.01	0.01	0.01	
Mean length of stay, d	4.02	5.17	5.43	4.53	
Mean charge, $	28,225.17	32,018.64	33,067.90	29,937.95	
Mean cost, $	8,090.41	9,097.29	9,832.92	8,589.50	
**Hispanic**					
n (%)	262,448 (73.93)	77,565 (21.85)	14,985 (4.22)	354,998 (100.00)	
Mortality rate	0.00	0.01	0.01	0.01	
Mean length of stay, d	3.54	5.02	5.36	3.94	
Mean charge, $	32,328.97	38,678.41	43,519.81	34,183.84	
Mean cost, $	8,518.59	9,898.87	10,984.73	8,923.22	
**Asian/Pacific Islander**					
n (%)	38,944 (75.83)	10,754 (20.94)	1,660 (3.23)	51,358 (100.00)	
Mortality rate	0.01	0.01	0.01	0.01	
Mean length of stay, d	3.84	5.93	6.40	4.36	
Mean charge, $	36,230.32	51,448.43	55,025.04	39,983.62	
Mean cost, $	9,762.20	13,694.84	14,330.96	10,723.20	
**Native American**					
n (%)	16,149 (62.46)	7,869 (30.43)	1,838 (7.11)	25,856 (100.00)	
Mortality rate	0.01	0.01	0.01	0.01	
Mean length of stay, d	3.60	5.34	5.05	4.23	
Mean charge, $	24,601.77	28,011.90	28,250.57	25,901.55	
Mean cost, $	8,381.63	9,448.44	9,081.64	8,756.74	
**Women aged 45–64 y**					
**White**					
n (%)	1,186,996 (36.45)	1,335,870 (41.03)	733,201 (22.52)	3,256,067 (100.00)	
Mortality rate	0.01	0.01	0.01	0.01	
Mean length of stay, d	4.13	4.84	5.12	4.65	
Mean charge, $	33,859.10	35,445.57	36,614.66	35,129.71	
Mean cost, $	10,554.25	11,064.03	11,351.18	10,942.63	
**Black**					
n (%)	198,781 (24.01)	359,803 (43.45)	269,462 (32.54)	828,046 (100.00)	
Mortality rate	0.02	0.02	0.02	0.02	
Mean length of stay, d	4.79	5.43	5.60	5.33	
Mean charge, $	36,747.47	38,743.52	38,706.02	38,252.29	
Mean cost, $	10,416.47	10,908.23	11,161.01	10,872.44	
**Hispanic**					
n (%)	150,184 (36.90)	162,767 (39.99)	94,051 (23.11)	407,002 (100.00)	
Mortality rate	0.01	0.01	0.02	0.01	
Mean length of stay, d	4.08	4.94	5.37	4.72	
Mean charge, $	38,043.64	43,204.39	46,865.87	42,145.14	
Mean cost, $	9,747.41	10,866.26	11,670.82	10,639.10	
**Asian/Pacific Islander**					
n (%)	34,958 (42.26)	32,206 (38.93)	15,562 (18.81)	82,725 (100.00)	
Mortality rate	0.02	0.02	0.02	0.02	
Mean length of stay, d	4.25	5.23	5.60	4.88	
Mean charge, $	44,425.20	52,586.01	59,232.48	50,306.06	
Mean cost, $	11,829.17	13,621.52	15,001.78	13,106.22	
**Native American**					
n (%)	11,639 (30.74)	15,921 (42.06)	10,298 (27.20)	37,858 (100.00)	
Mortality rate	0.02	0.01	0.02	0.01	
Mean length of stay, d	4.19	4.80	5.00	4.66	
Mean charge, $	30,139.27	32,174.50	34,456.78	32,167.51	
Mean cost, $	10,441.54	10,864.81	10,912.14	10,747.29	
**Women aged ≥65 y**					
**White**					
n (%)	829,817 (13.71)	2,456,096 (40.58)	2,766,399 (45.71)	6,052,312 (100.00)	
Mortality rate	0.04	0.03	0.04	0.04	
Mean length of stay, d	5.18	5.19	5.41	5.29	
Mean charge, $	35,414.11	33,773.19	33,429.61	33,842.53	
Mean cost, $	11,057.53	10,506.00	10,386.20	10,527.33	
**Black**					
n (%)	68,195 (9.19)	291,036 (39.22)	382,811 (51.59)	742,042 (100.00)	
Mortality rate	0.06	0.04	0.03	0.04	
Mean length of stay, d	6.71	6.10	5.98	6.10	
Mean charge, $	48,248.46	41,551.23	38,663.57	40,683.88	
Mean cost, $	12,980.58	11,515.06	11,113.04	11,443.54	
**Hispanic**					
n (%)	69,704 (14.14)	206,336 (41.86)	216,909 (44.00)	492,949 (100.00)	
Mortality rate	0.04	0.03	0.03	0.03	
Mean length of stay, d	5.14	5.35	5.83	5.53	
Mean charge, $	44,228.99	45,217.73	49,240.79	46,840.52	
Mean cost, $	10,717.20	10,871.76	11,856.34	11,281.33	
**Asian/Pacific Islander**					
n (%)	21,015 (14.31)	61,799 (42.07)	64,088 (43.63)	146,902 (100.00)	
Mortality rate	0.05	0.04	0.04	0.04	
Mean length of stay, d	5.70	5.53	5.89	5.71	
Mean charge, $	54,013.97	52,729.35	59,779.75	55,956.26	
Mean cost, $	13,741.25	12,850.96	14,155.06	13,542.71	
**Native American**					
n (%)	6,502 (15.77)	16,773 (40.67)	17,962 (43.56)	41,236 (100.00)	
Mortality rate	0.05	0.03	0.03	0.03	
Mean length of stay, d	4.95	5.06	5.25	5.13	
Mean charge, $	30,440.56	32,409.60	34,475.43	32,996.89	
Mean cost, $	11,157.61	11,059.78	10,675.72	10,908.19	

We identified the most common pairs of conditions nested within age and sex for adults discharged with 2 or more conditions (Table 4). For example, an estimated 1,044,459 adult men aged 18 through 44 have 2 or more chronic conditions on their discharge abstract. Of those, approximately 24% have the dyad of depression and substance abuse. Clearly, hypertension is found in most of these combinations. After age 44, two-way combinations of coronary artery disease, diabetes, hyperlipidemia, and hypertension are the most prominent dyads.

**Table 4:** Five Most Prevalent Chronic Condition Dyads for US Adults With 2 or More Chronic Conditions, by Sex and Age, Nationwide Inpatient Sample, 2009

Sex, Age, and Dyad	%^a^
**Men**	
**18–44 y (n = 1,044,459)**	
Depression/substance abuse	23.9
Hypertension/diabetes	17.8
Hyperlipidemia/hypertension	15.1
Hypertension/substance abuse	13.6
Hypertension/depression	11.9
**45–64 y (n = 3,420,573)**	
Hypertension/hyperlipidemia	31.8
Hypertension/diabetes	29.5
Hypertension/coronary artery disease	26.1
Hyperlipidemia/coronary artery disease	19.5
Diabetes/hyperlipidemia	16.6
**≥65 y (n = 5,103,409**)	
Hypertension/coronary artery disease	37.1
Hypertension/hyperlipidemia	34.2
Hypertension/cardiac arrhythmia	28.8
Hypertension/diabetes	27.9
Hyperlipidemia/coronary artery disease	24.9
**Women**	
**18–44 y (n = 987,310)**	
Depression/substance abuse	22.0
Hypertension/diabetes	18.6
Hypertension/depression	15.7
Depression/asthma	12.5
Hyperlipidemia/hypertension	11.3
**45–64 y (n = 3,150,679)**	
Hypertension/diabetes	31.3
Hyperlipidemia/hypertension	28.3
Hypertension/depression	18.0
Hyperlipidemia/diabetes	16.7
Hypertension/coronary artery disease	16.0
**≥65 y (n = 6,618,736)**	
Hypertension/hyperlipidemia	32.6
Hypertension/coronary artery disease	26.9
Hypertension/diabetes	26.7
Hypertension/cardiac arrhythmia	25.9
Hypertension/congestive heart failure	19.2

a Percentage does not total 100% because the list presents only the top-ranked disease groupings.

Triads of chronic conditions reveal a few additional conditions beyond those demonstrated within the most common dyads (Table 5). We determined the 5 most common triads of the 15 chronic condition groups, nested within age and sex. Chronic kidney disease and then, after age 44, cardiac arrhythmia make their way into the most frequent triads.

**Table 5:** Five Most Prevalent Chronic Condition Triads for US Adults With 3 or More Chronic Conditions, by Sex and Age, Nationwide Inpatient Sample, 2009

Sex, Age, and Triad	%^a^
**Men**	
**18–44 y (n = 481,305)**	
Diabetes/hyperlipidemia/hypertension	14.3
Hypertension/depression/substance abuse	10.7
Diabetes/hyperlipidemia/chronic kidney disease	10.0
Hypertension/hyperlipidemia/coronary artery disease	9.2
Diabetes/hypertension/depression	6.8
**45–64 y (n = 2,359,061)**	
Hypertension/hyperlipidemia/coronary artery disease	22.7
Diabetes/hyperlipidemia/hypertension	20.1
Diabetes/hypertension/coronary artery disease	16.7
Hyperlipidemia/coronary artery disease/diabetes	11.7
Diabetes/hypertension/chronic kidney disease	11.0
**≥65 y (n = 4,123,675)**	
Hypertension/hyperlipidemia/coronary artery disease	25.1
Hypertension/coronary artery disease/cardiac arrhythmia	20.3
Diabetes/hypertension/coronary artery disease	18.2
Diabetes/hyperlipidemia/hypertension	16.7
Hyperlipidemia/hypertension/cardiac arrhythmia	16.2
**Women**	
**18–44 y (n = 429,490)**	
Diabetes/hyperlipidemia/hypertension	13.6
Diabetes/hyperlipidemia/hypertension	11.5
Diabetes/hypertension/depression	9.9
Diabetes/hypertension/chronic kidney disease	7.9
Hypertension/hyperlipidemia/depression	7.7
**45–64 y (n = 2,075,306)**	
Diabetes/hyperlipidemia/hypertension	22.7
Hypertension/hyperlipidemia/coronary artery disease	21.0
Diabetes/hypertension/coronary artery disease	13.6
Hypertension/hyperlipidemia/depression	13.2
Diabetes/hypertension/chronic kidney disease	10.4
**≥65 y (n = 5,208,808)**	
Hypertension/hyperlipidemia/coronary artery disease	16.9
Diabetes/hyperlipidemia/hypertension	15.2
Hypertension/coronary artery disease/cardiac arrhythmia	13.5
Diabetes/hypertension/coronary artery disease	13.2
Hyperlipidemia/hypertension/cardiac arrhythmia	13.0

a Percentage does not total 100% because the list presents only the top-ranked disease groupings.

## Summary

An estimated 20 million adult discharges from community hospitals in the United States have 2 or more chronic conditions noted on their hospital record. These discharges include nearly 66% of all adult discharges from US hospitals. More than 9 million adult discharges (almost a third of all discharges) are estimated to have 4 or more chronic conditions. These data demonstrate compelling findings to support the fourth goal of the HHS Multiple Chronic Conditions Strategic Framework by providing the detailed distribution of multiple chronic conditions among adult discharges from community hospitals. The data suggest that payer group and racial/ethnic groups are associated with the number of chronic conditions listed in a hospital discharge summary, as are cost per stay, mortality rate, stays per year, and cost per year. Although the causal underpinnings of the associations are not explored here, the differences in cost, length of stay, and mortality for patients with 4 or more chronic conditions are substantial compared with those for adults with 1 chronic condition or none. Given that hospital costs remain the largest component of health care spending, the concentration of use and cost among patients with MCC demonstrates the need for a sustained effort to identify and treat MCC. Many factors could influence the incidence and management of chronic illness that are confounded with demographic and payer categories or operate differently in different groups.

Although the HCUP NIS is a singular source for national estimates of all-payer hospital-based use, outcomes, and cost, this database has strengths and limitations for examining MCCs. For example, while the HCUP NIS includes use and cost for private, public, and uninsured patients, it is limited to the experience of hospitalized adults. The data do not include outpatient costs or physician costs associated with MCC treatment. In addition, the data are not at the patient level but at the discharge level, so that use of frequently readmitted patients to the hospital is included in the database.

An increased number of these 15 chronic conditions for any hospitalized adult is associated with higher cost per stay and higher mortality. The well-known association between increasing age and number of chronic conditions is demonstrated in our study as well. However, nesting payer or racial/ethnic groups within age and sex highlights important associations by age and sex. These findings may help public health agencies and private health plans to identify subpopulations that will have higher costs and poorer outcomes. This information might be used in designing and targeting new services, patient education, or financial incentives to support effective management of complex chronic illness. Once implemented, these data can also help evaluate the impact of new clinical or delivery system strategies on hospital use, outcomes, or cost.

## Appendix. Coding for Selected Chronic Conditions

**Table:**

Condition	CCS Category or Cluster
Hypertension	98, 99
Hyperlipidemia	53
Congestive heart failure	108
Coronary artery disease (CAD) (includes acute myocardial infarction, which indicates chronic underlying CAD)	100, 101
Diabetes	49, 50
Stroke (includes acute stroke but indicates underlying cerebrovascular disease)	109–112
Cardiac arrhythmias	105, 106
Arthritis	202, 203
Cancer	11–43
Depression	657
Dementia (includes Alzheimer’s and other senile dementias)	653
Substance abuse disorders	660, 661
Chronic obstructive pulmonary disease	127
Asthma	128
Chronic kidney disease	156, 158
HIV	5
Hepatitis	6
Autism spectrum disorder	ICD-9-CM 29900, 29901
Schizophrenia	659
Osteoporosis	206
