# Erratum, Vol. 10, April 25 Release

**DOI:** 10.5888/pcd10.120292e

**Published:** 2013-05-30

In the article “Hospital Utilization, Costs, and Mortality for Adults With Multiple Chronic Conditions, Nationwide Inpatient Sample, 2009,” an entry in Table 3 gave an incorrect figure. Mean costs for Asian/Pacific Islander men aged 65 and older with 4 or more chronic conditions should be $16,261.18, not $16,61.18.

The correction was made to our website on May 20, 2013, and appears online at http://www.cdc.gov/pcd/issues/2013/12_0292.htm. We regret any confusion or inconvenience this error may have caused.

